# Author Correction: Transition from simple to complex contagion in collective decision-making

**DOI:** 10.1038/s41467-022-30552-9

**Published:** 2022-05-12

**Authors:** Nikolaj Horsevad, David Mateo, Robert E. Kooij, Alain Barrat, Roland Bouffanais

**Affiliations:** 1 University of Ottawa, Ottawa, Canada; 2 Kido Dynamics, Lausanne, Switzerland; 3 Delft University of Technology, Delft, The Netherlands; 4 The Netherlands Organization for Applied Scientific Research (TNO), The Hague, The Netherlands; 5 Aix Marseille Univ, Université de Toulon, CNRS, CPT, Turing Center for Living Systems, Marseille, France; 6 Tokyo Tech World Research Hub Initiative (WRHI), Tokyo Institute of Technology, Tokyo, Japan

**Keywords:** Complex networks, Nonlinear phenomena

Correction to: *Nature Communications* 10.1038/s41467-022-28958-6, published online 17 March 2022.

The original version of this Article contained an error in the Abstract, which incorrectly read: ‘Here, we show theoretically, and experimentally with a multi-robot system, that such a transition from simple to complex contagion can also bed observed in an archetypal model of distributed decision-making devoid of any thresholds or nonlinearities.’

The correct form of the fourth sentence in the Abstract is:

‘Here, we show theoretically, and experimentally with a multi-robot system, that such a transition from simple to complex contagion can also be observed in an archetypal model of distributed decision-making devoid of any thresholds or nonlinearities.’

