# Supplementary material for: Transition from simple to complex contagion in collective decision-making
Source: Nat Commun. 2022 Mar 17;13:1442. doi: 10.1038/s41467-022-28958-6 (PMC8931172; doi:10.1038/s41467-022-28958-6)
Supplement: Supplementary file 1 — Supplementary Information [file 41467_2022_28958_MOESM1_ESM.pdf]

Supplementary Information: Transition from simple to complex contagion in collective decision-making

Nikolaj Horsevad[1,*], David Mateo[2], Robert Kooij[3], Alain Barrat[4,5], and Roland Bouffanais[6,†]

[1]Singapore University of Technology and Design, Singapore, Singapore
[2]Kido Dynamics, Lausanne, Switzerland
[3]Delft University of Technology, Delft, The Netherlands
[4]Aix Marseille Univ, Université de Toulon, CNRS, CPT, Turing Center for Living Systems, Marseille, France
[5]Tokyo Tech World Research Hub Initiative (WRHI), Tokyo Institute of Technology, Tokyo, Japan
[6]University of Ottawa, Ottawa, Canada
[*]nikolajhorsevad@gmail.com
[†]roland.bouffanais@uottawa.ca

Polarization speed in threshold models

As mentioned in the main text, a useful metric to analyze the speed of contagion is given by the so-called polarization speed v, which measures the speed at which a random activator node and its neighbors can in turn activate a given fraction of nodes.

Influence of the fraction of activated nodes

In the main text (Fig. 1), the results are generated and shown when v is defined as the speed to reach a fraction of 30% activated nodes. Figure S1 shows the same results for nine different fractions of activated nodes—ranging from 10% all the way to 90%, by increment of 10%, and including 30% for the sake of comparison.

Influence of the system size on polarization speed

In the main text (Fig. 1), the results are generated and shown when v is defined as the speed to reach a fraction of 30% activated nodes, with $N = 10,000$ nodes. In Figure S1 we observe the transition threshold slightly changing with the cascade size. Here we show in Figure S2 the same result, but with $N = 5,000$, to show the impact of network size. Qualitatively there is no significant change to the results when halving the systemsize.

Distributions of polarization speed with respect to the network metrics

To generate Fig. 1(b), we calculate the Spearman's correlation coefficient r_s between the polarization speed v and each network property $\chi \in \{C, \ell, R_g\}$, for each threshold value θ (see Methods). Figure S3 provides the actual distributions of the polarization speed v with respect to χ for three distinct values of the threshold: $\theta = 0.08$ (simple contagion), $\theta = 0.24$ (transition region), and $\theta = 0.3$ (complex contagion).

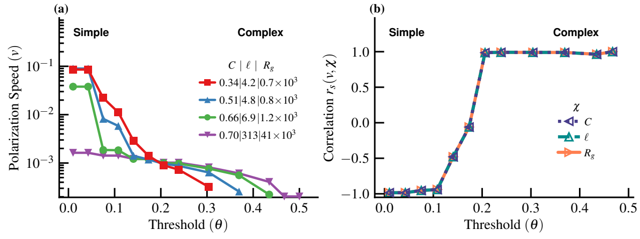

(a) Cascade size: 10% activated nodes

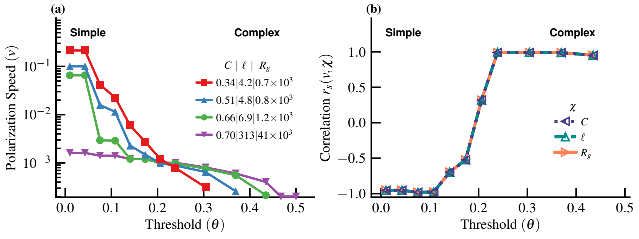

(b) Cascade size: 20% activated nodes

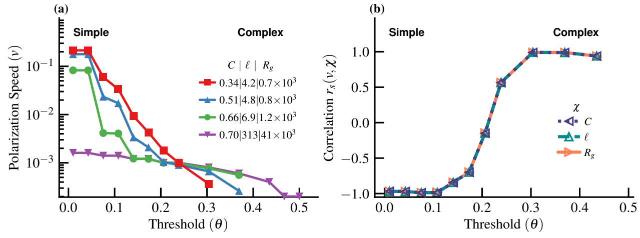

(c) Cascade size: 30% activated nodes

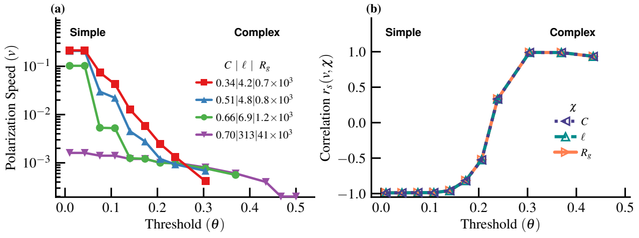

(d) Cascade size: 40% activated nodes

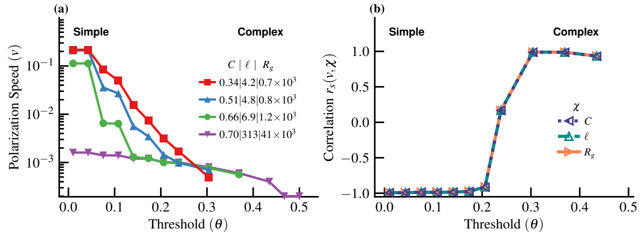

(e) Cascade size: 50% activated nodes

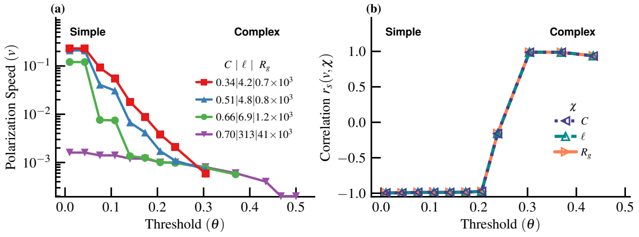

(f) Cascade size: 60% activated nodes

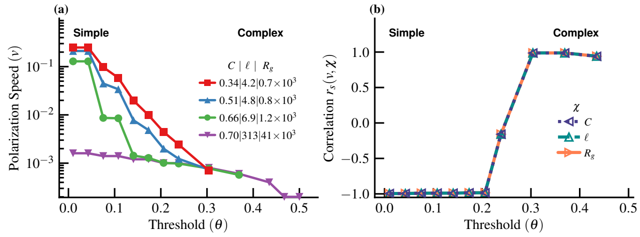

(g) Cascade size: 70% activated nodes

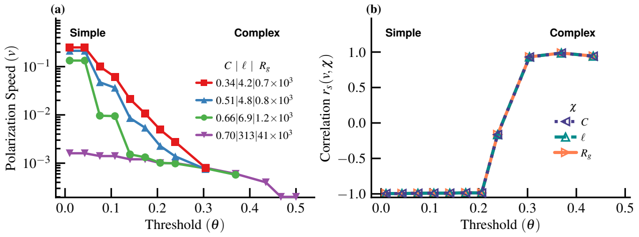

(h) Cascade size: 80% activated nodes

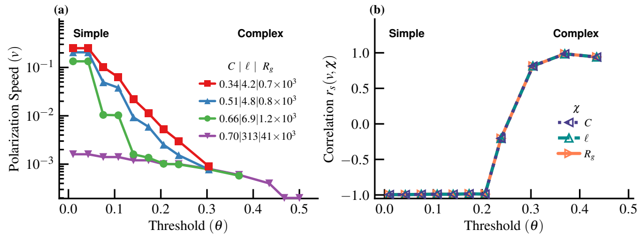

(i) Cascade size: 90% activated nodes

Figure S1. Linear threshold model on WS networks of $N = 10,000$ nodes, with fixed average degree $\langle k \rangle = 16$ and uniform threshold θ. Initially a single randomly selected seed node and its neighbors are activated. The WS rewiring probability p is used to generate network samples having specific values of $\chi \in \{C, \ell, R_g\}$. Spearman's correlation coefficients r_s between the polarization speed v and each network metric $\chi \in \{C, \ell, R_g\}$ are also shown for different cascade sizes.

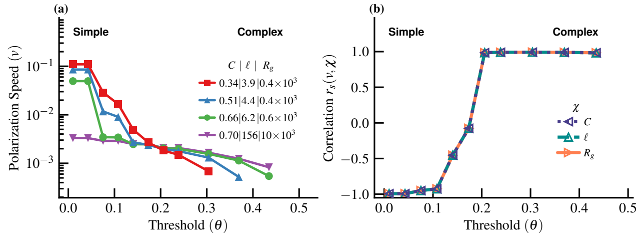

(a) Cascade size: 10% activated nodes

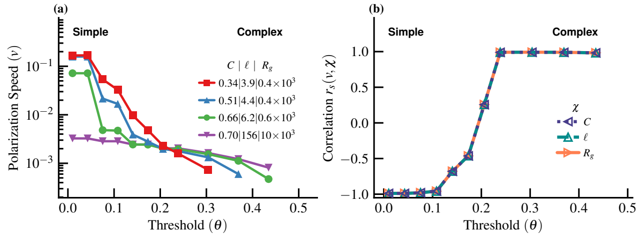

(b) Cascade size: 20% activated nodes

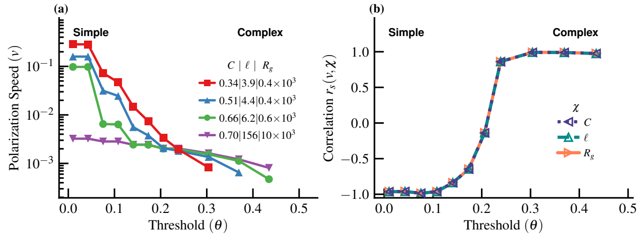

(c) Cascade size: 30% activated nodes

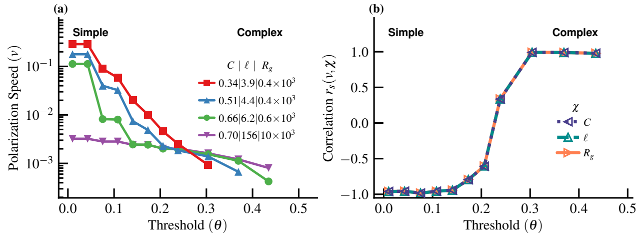

(d) Cascade size: 40% activated nodes

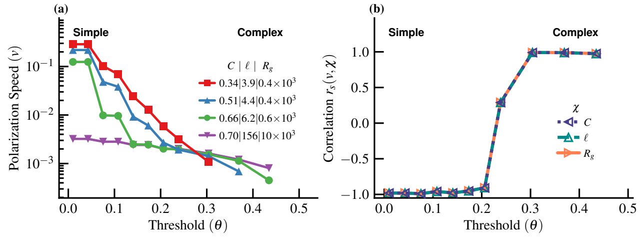

(e) Cascade size: 50% activated nodes

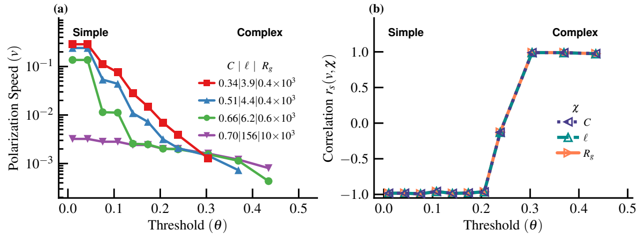

(f) Cascade size: 60% activated nodes

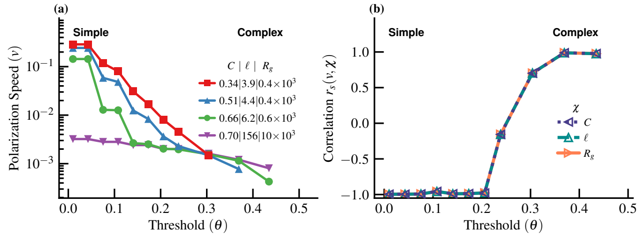

(g) Cascade size: 70% activated nodes

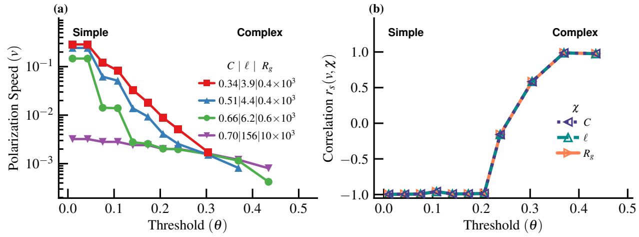

(h) Cascade size: 80% activated nodes

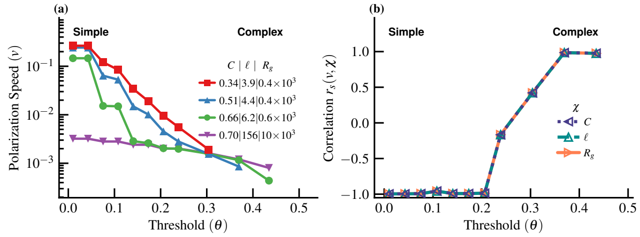

(i) Cascade size: 90% activated nodes

Figure S2. Linear threshold model on WS networks of $N = 5,000$ nodes, with fixed average degree $\langle k \rangle = 16$ and uniform threshold θ. Initially a single randomly selected seed node and its neighbors are activated. The WS rewiring probability p is used to generate network samples having specific values of $\chi \in \{C, \ell, R_g\}$. Spearman's correlation coefficients r_s between the polarization speed v and each network metric $\chi \in \{C, \ell, R_g\}$ are also shown for different cascade sizes.

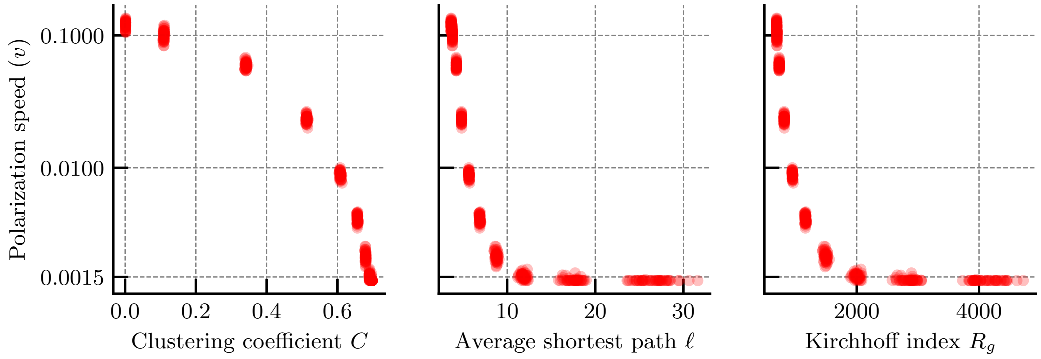

(a) Distributions of polarization speeds used in Fig. 1(b) for $\theta = 0.08$, and corresponding to $r_s \approx -1$.

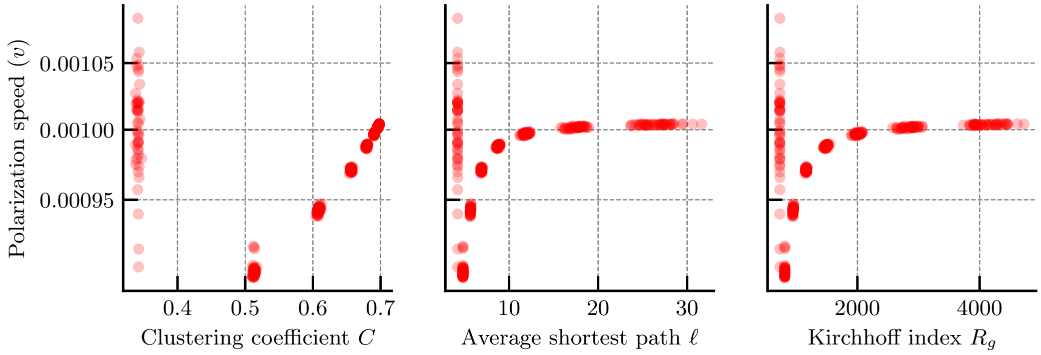

(b) Distributions of polarization speeds used in Fig. 1(b) for $\theta = 0.24$, and corresponding to $r_s \approx 0$.

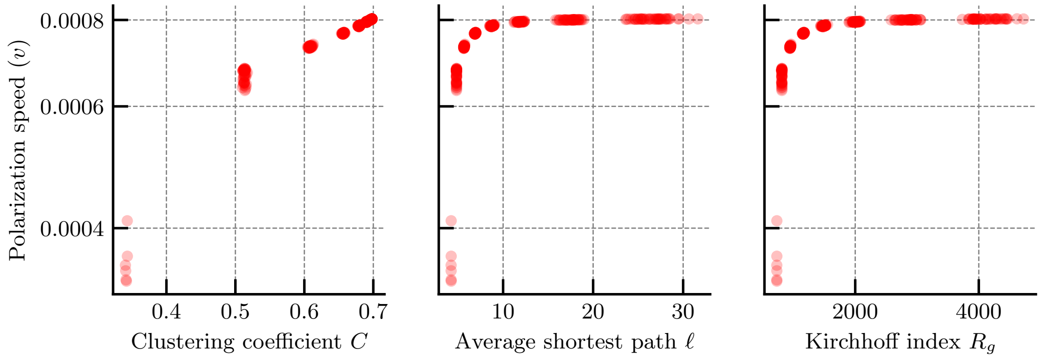

(c) Distributions of polarization speeds used in Fig. 1(b) for $\theta = 0.3$, and corresponding to $r_s \approx 1$.

Figure S3. Distributions of polarization speeds used in Fig. 1(b). Note that the y-axis uses a logarithmic scale like other figures with the polarization speed.

Transition from simple to complex contagion in consensus models

Influence of the sample of networks on the Spearman's correlation coefficient

In the main text (Fig. 2(c)), we show the Spearman's correlation coefficient r_s between $\bar{H}^2(\omega)$ and $\bar{\chi} \in \{\bar{C}, \bar{\ell}, \bar{R}_g\}$. It is shown that for these three network metrics, r_s exhibits a clear sigmoidal trend from -1 at low frequency to $+1$ at high frequency. Additional WS networks—with different values of the average degree—are selected to form a subsample of these networks having uncorrelated network metrics (Methods). Given this extended network sampling (green dots in the insert of Fig. 2(c)), we obtain the following key result: at low frequency, $\bar{H}^2$ is highly (negatively) correlated with $\bar{R}_g$ and practically uncorrelated with $\bar{C}$, while the opposite is true at high frequency.

Here, we consider the influence of the choice of this subsample of networks by showing the same results for two different subsamples: (a) narrowing the range of values for the Kirchhoff index $\bar{R}_g$ (Fig. S4a), and (b) extending the range of $\bar{R}_g$ values (Fig. S4b).

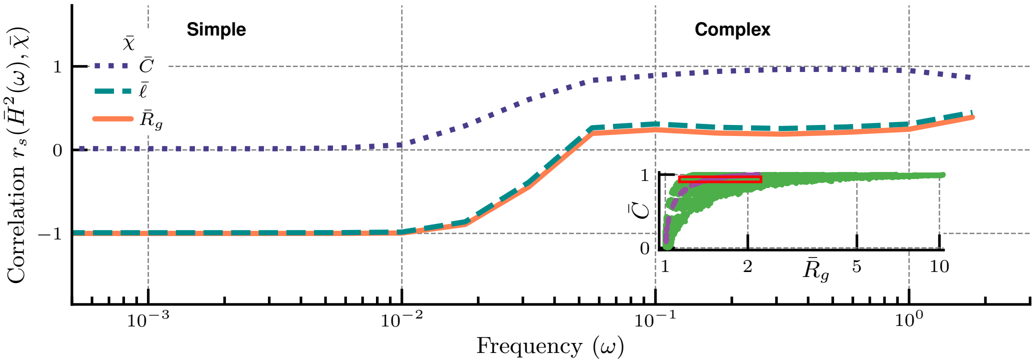

(a) Narrow range of normalized Kirchhoff index, corresponding to the network samples within the red rectangular box.

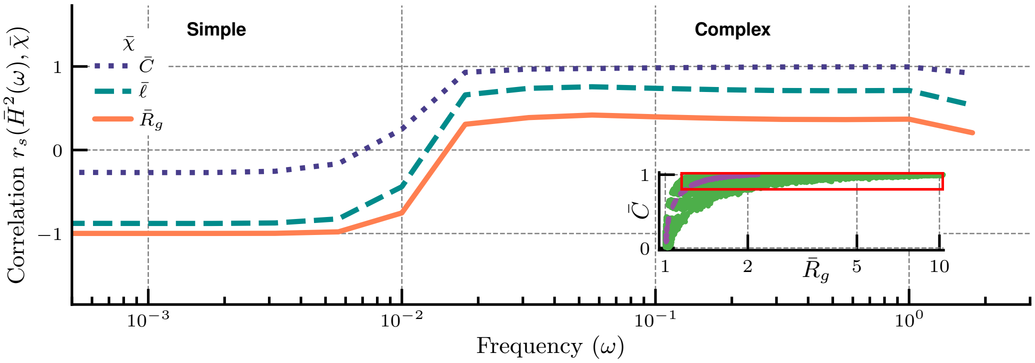

(b) Extended range of normalized Kirchhoff index, corresponding to the network samples within the red rectangular box.

Figure S4. Spearman's correlation coefficient r_s between normalized collective response $\bar{H}^2$ and the normalized network metrics $\bar{\chi} \in \{\bar{C}, \bar{\ell}, \bar{R}_g\}$. Same figure as Fig.2(b-c) in the main text, but with different subsamples of networks owing to the different range of values for the network metrics.

Influence of the system size on collective gain

Similarly to Figure S2, we should consider the effects of system size on the transition observed in the Leader-Follower consensus dynamics. In Fig. 2 in the main text we have $N = 240$, here we present Figure S5 with $N = 120$ nodes. Obviously the range of the network metrics will shift when decreasing the systems size, especially the distance metrics, so the subsample has to be adjusted accordingly. But after an uncorrelated subsample is chosen, the correlations observed in Figure S5 are equivalent to the larger system size. One notable difference observed is the transition happens at a higher frequency, which is entirely consistent with previous work in[1], and the effects of the complex contagion at high frequency are less pronounced in panel (a).

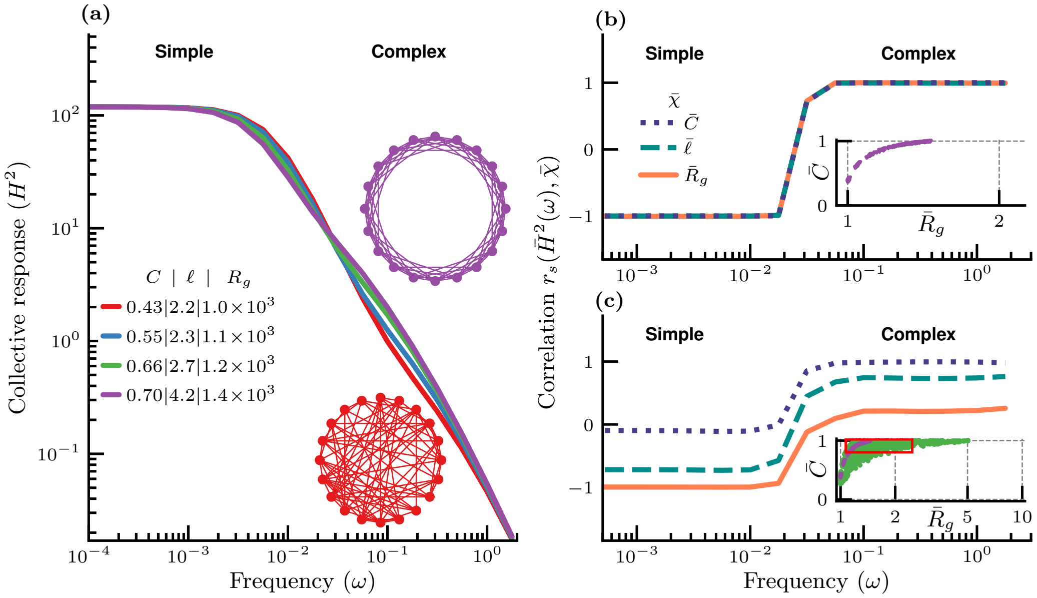

Figure S5. Spearman's correlation coefficient r_s between normalized collective response $\bar{H}^2$ and the normalized network metrics $\bar{\chi} \in \{\bar{C}, \bar{\ell}, \bar{R}_g\}$. Same figure as Fig.2(b-c) in the main text, but with different subsamples of networks owing to the different range of values for the network metrics. $N = 120$

Influence of the network model on the Spearman's correlation coefficient

In the main text (Fig. 2(c)), we show the Spearman's correlation coefficient r_s between $\bar{H}^2(\omega)$ and $\bar{\chi} \in \{\bar{C}, \bar{\ell}, \bar{R}_g\}$. This is shown for the small-world WS family of networks. However, other networks that yield some meaningful variations in the relevant metrics can be used. For instance, here, we consider the influence of the choice of the network family by showing the same results for the modified Holme–Kim model[2] (Fig. S6). This particular model generates heterogeneous networks—i.e., with a scale-free degree distribution, where a single control parameter enables us to tune the clustering coefficient (insert of Fig. S6). We observe the exact same trends as in Fig. 2(b) with homogeneous networks.

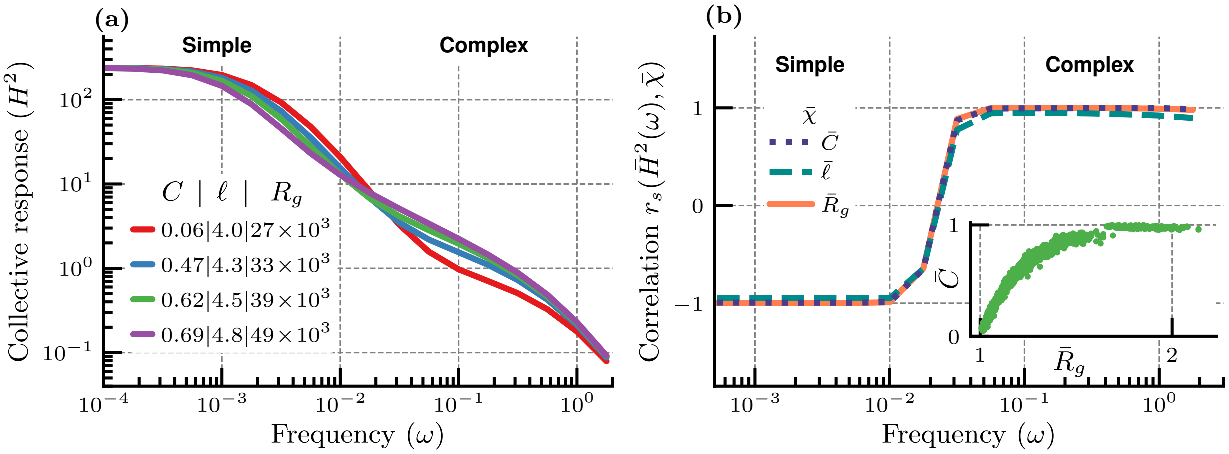

Figure S6. Correlation results obtained with scale-free networks generated by the modified Holme–Kim model[2].

Distribution of the normalized collective frequency response with respect to the normalized network metrics

In the main text (Fig. 2(c)), the Spearman's correlation results are generated and shown for the subsample of networks corresponding to the green dots in the insert of Fig. 2(c).

For completeness, we show here the actual dependence of the normalized collective frequency response $\bar{H}^2(\omega)$ with the normalized network metrics $\bar{\chi} \in \{\bar{C}, \bar{\ell}, \bar{R}_g\}$ for the full sample of networks, with varying average degree $\langle k \rangle$, for three selected frequencies: (a) low frequency $\omega = 0.0001$ (Fig. S7a) corresponding to a simple contagion, (b) $\omega = 0.0178$ (Fig. S7b) in the transition region, and (c) high frequency $\omega = 0.1$ (Fig. S7c) associated with a complex contagion.

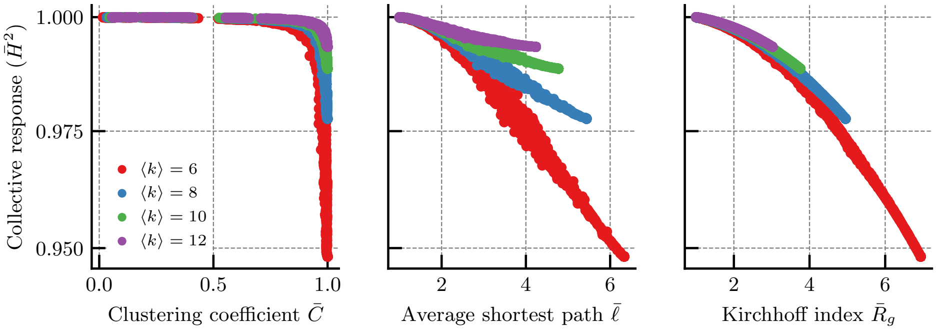

(a) Distributions at $\omega = 0.0001$, representative of the simple contagion region.

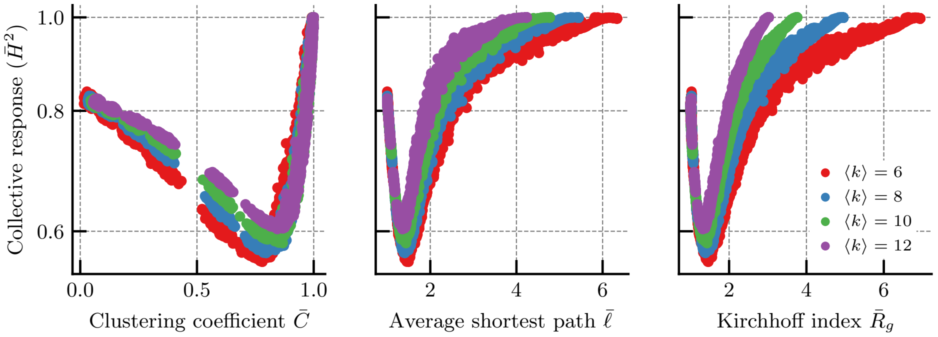

(b) Distributions at $\omega = 0.0178$, representative of the transition region.

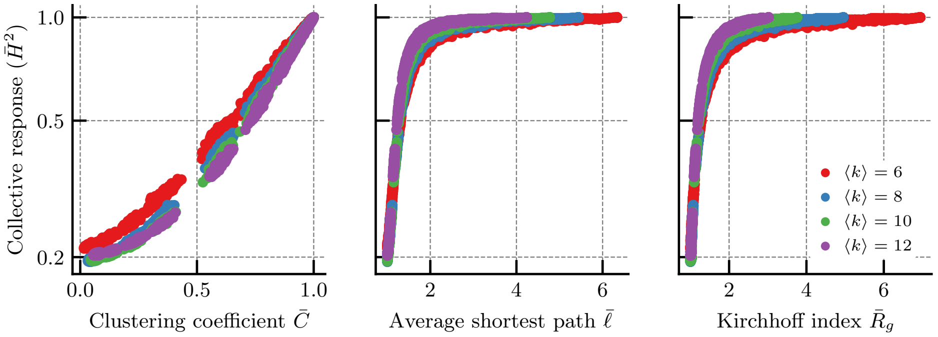

(c) Distributions at $\omega = 0.1$, representative of the complex contagion region.

Figure S7. Distributions of the normalized collective frequency response $\bar{H}^2(\omega)$ with the normalized metrics $\bar{\chi} \in \{\bar{C}, \bar{\ell}, \bar{R}_g\}$ used in Fig. 2(c).

Distribution of collective frequency response with respect to the network metrics

For completeness, we provide in Fig. S8 the same results as in Fig. S7 with *non-normalized* quantities.

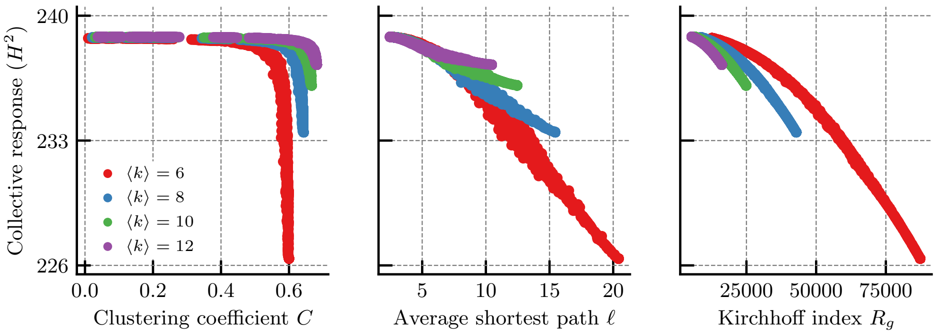

(a) Distributions at $\omega = 0.0001$, representative of the complex contagion region.

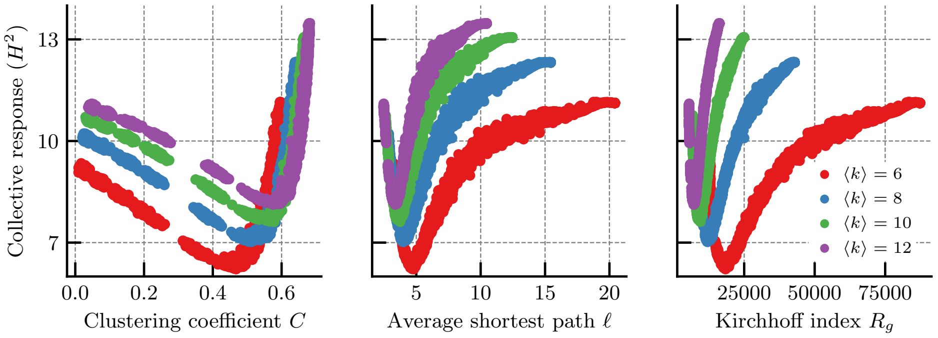

(b) Distribution at $\omega = 0.0178$, representative of the complex contagion region.

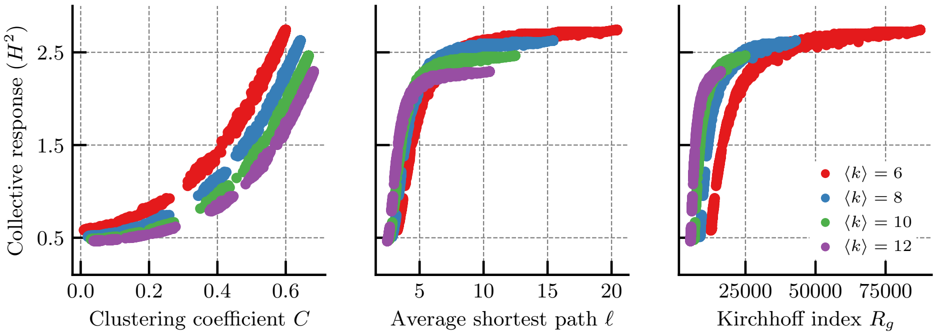

(c) Distribution at $\omega = 0.1$, representative of the complex contagion region.

Figure S8. Distributions of the *non-normalized* collective frequency response $H^2(\omega)$ with respect to the *non-normalized* network metrics $\chi \in \{C, \ell, R_g\}$, used in Fig. 2(c).

Distribution of ℓ and C

In Figure S9 we show the distribution of C and ℓ, for comparison with the inserts on Fig. 2 in the main text.

Schematic of the network between eBots

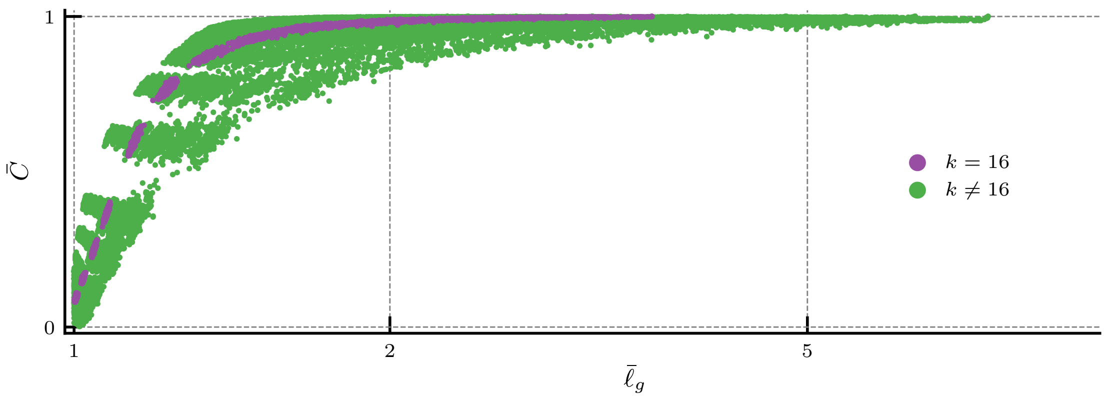

Figure S9. Distribution of $\bar{C}$ with $\bar{\ell}$. $k = \langle 16 \rangle$ is highlighted in purple, showing how for fixed $\langle k \rangle$ the clustering C increases monotonically with the average shortest path ℓ.

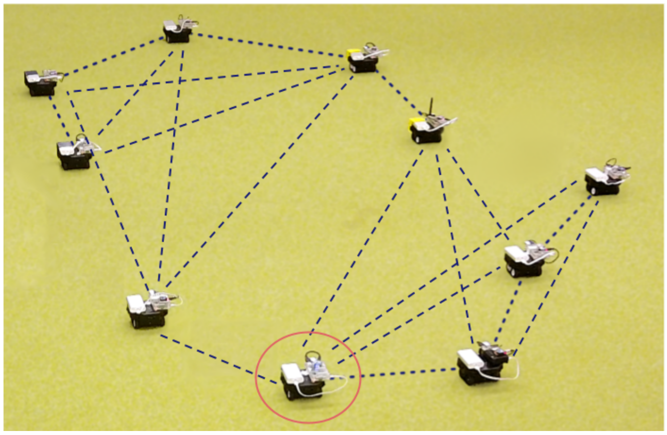

Figure S10. Snapshot of the $N = 10$ robotic units with the leader agent circled in red. The topology of the caveman network is superimposed with edges shown in dashed lines.

Supplementary References

1. Mateo, D., Horsevad, N., Hassani, V., Chamanbaz, M. & Bouffanais, R. Optimal network topology for responsive collective behavior. *Sci. Adv.* **5**, eaau0999, DOI: http://doi.org/10.1126/sciadv.aau0999 (2019).

2. Sekunda, A., Komareji, M. & Bouffanais, R. Interplay between signaling network design and swarm dynamics. *Netw. Sci.* **4**, 244–265, DOI: http://doi.org/10.1017/nws.2016.5 (2016).
